# Blood biomarkers for canine cancer, from human to veterinary oncology

**DOI:** 10.1111/vco.12848

**Published:** 2022-07-20

**Authors:** Philippe Colombe, Jérémy Béguin, Ghita Benchekroun, Delphine Le Roux

**Affiliations:** ^1^ Ecole Nationale Vétérinaire d'Alfort BioPôle Alfort Maisons‐Alfort France; ^2^ Ecole Nationale Vétérinaire d'Alfort CHUVA, Service de Médecine Interne Maisons‐Alfort France; ^3^ Anses, INRAE, Ecole Nationale Vétérinaire d'Alfort UMR VIROLOGIE, Laboratoire de Santé Animale Maisons‐Alfort France; ^4^ Ecole nationale Vétérinaire d'Alfort Univ Paris Est Créteil, INSERM, IMRB Maisons‐Alfort France; ^5^ Anses, INRAE, Ecole Nationale Vétérinaire d'Alfort UMR BIPAR, Laboratoire de Santé Animale Maisons‐Alfort France

**Keywords:** biomarker, cancer, liquid biopsy, veterinary

## Abstract

In recent decades, interest in circulating tumour biomarkers is increasing both in human and veterinary oncology. An ideal tumour biomarker would allow early diagnosis of neoplasia, identify it specifically, accurately, establish a prognosis and predict its behaviour, especially regarding different therapeutic solutions. It would also allow to monitor its evolution over time and all this in a non‐invasive and inexpensive way. Actually, no biomarkers meeting all of these criteria have been identified in veterinary medicine, particularly due to a lack of specificity of the main protein tumour biomarkers studied to date. However, great hope is currently placed in biomarkers grouped under the name of liquid biopsy, which could prove to be effective tools for common clinical use in the near future. This review gives an update on blood cancer biomarkers studied in dogs, such as ions, proteins, nucleic acids and also circulating cells, of which some might become more prominent in the coming years to help improve the management of animal care.

## 1 INTRODUCTION

The use of biomarkers represents a major challenge in the field of oncology in both human and veterinary medicine. The growing knowledge of biomarkers over the last two decades has enabled better management of patients with tumours. Ideally a tumour biomarker would be easy to measure, should have perfect sensitivity and specificity, with the aim of perfectly differentiating individuals suffering from a neoplastic process from healthy individuals, and at the earliest possible stages. It should also be able to identify a subject affected by any tumour process, allow monitoring of the evolution of cancer over time and forecasting of relapses or recurrences.^1^

The vascular compartment is easily accessible, so blood is a sample of choice to identify and measure tumour biomarkers. Indeed, the sampling procedure is minimally invasive and the measurement of one (or more) component is often quick. In contrast, tumour biopsies are more invasive and often take longer to analyse and interpret. Moreover, in veterinary medicine, tumour biopsies, by requiring sedation or general anaesthesia, increase the owner's expenses as well as exposure of the animal to additional risks such as dissemination of tumour cells, enlargement of the surgical site, and possible post‐anaesthetic complications. Furthermore, unlike the majority of tumour biopsies, blood sampling can be repeated at close intervals, which allows monitoring of parameters over time and therefore assessment of potential changes in cancer behaviour, with or without treatment. There are many types of blood biomarkers: ions, proteins, carbohydrates, lipids, nucleic acids and also cells. However, none of them is yet commonly used for diagnostic purposes in veterinary medicine. Instead of focusing on one single category of biomarker or all biomarkers in one single form of cancer, this review provides an overview of the scientific advances concerning selected blood parameters of various type, that have shown potential interest as biomarkers for different canine cancers, inspired from extensive studies in human oncology, the latter being excluded from this manuscript as reviewed elsewhere.^2^

## 2 IONS AS BIOMARKERS

### 2.1 The copper ion and copper isotopes

Copper ion (Cu^2+^) is an essential micronutrient, with involvement in many fundamental physiological processes. In humans, copper has also been involved in the processes of tumorigenesis, angiogenesis, tumour migration and metastasis development^3^, ^4^, ^5^, ^6^ but only a few animal studies have focused on this topic. However, one study demonstrated a two‐fold increase in Cu^2+^ concentrations in bitches with mammary tumours of various types compared with healthy bitches.^7^ Unfortunately, the small size of this study and the lack of comparison of nutrient intake, precludes for the moment, the possibility to use serum Cu^2+^ as a diagnostic biomarker for mammary tumours in dogs.

New mass spectrometry methods such as multi‐collector inductively coupled plasma‐mass spectometry (MC‐ICP‐MS), allowed the measurement of copper isotopes ^65^Cu/^63^Cu ratio (δCu) in dogs' blood. Blood δCu measured in dogs with neoplastic or non‐neoplastic disease was significantly lower compared with healthy dogs. However, δCu levels between neoplastic and non‐neoplastic dogs were not significantly different. In addition, following a chemotherapy protocol for lymphoma, an increase of the δCu was seen in five out of six dogs in clinical remission.^8^ Interestingly, to measure these copper ions, whole blood can be frozen at −20°C and processed once defrosted, to purify copper elements before performing MC‐ICP‐MS, which is therefore a technical advantage to investigate larger cohorts. Indeed, to show a possible interest of the δCu in monitoring the response to chemotherapy treatment in dogs, these larger studies are now required, with a focus on specific neoplastic conditions to identify those that may present significant isotope variation compared with other non‐neoplastic conditions.

### 2.2 The zinc ion

Only two published studies have focused on measuring Zinc ion (Zn^2+^) serum concentration in neoplastic and healthy dogs. A significant decrease in Zn^2+^ serum concentrations was observed in 50 dogs diagnosed with IIIa or IVa lymphoma (classification according to the World Health Organisation's) compared with 50 healthy dogs. This decrease was also seen in dogs suffering from osteosarcoma but the difference with the healthy dogs was not significant.^9^ A significant decrease in serum Zn^2+^ concentration was also observed in bitches with mammary tumours compared with healthy bitches.^7^ Since these two studies from the past decades, Zn^2+^ serum levels have not been further studied in other tumour types or with bigger dog cohorts. This could be attributed to the fact that dietary intake may influence Zn^2+^ serum levels, which could be hard to monitor in animals. Yet, a lot of studies in human oncology have shown a significant decrease in serum Zn^2+^ concentration in many cancers and some also suggest the potential benefit of Zn^2+^ supplementation during therapy.^10^, ^11^, ^12^, ^13^, ^14^ This should justify and motivate more research on Zn^2+^ blood levels in the veterinary oncology field since it is now easily measurable in serum with a colorimetric assay.^12^

## 3 PROTEINS AS BIOMARKERS

### 3.1 The carcino‐embryonic antigen

The carcino‐embryogenic antigen (CEA) is a membrane glycoprotein, mainly synthesised in certain portions of the digestive tract and plays a role in cell adhesion. CEA blood concentration is very low under physiological conditions but some tumour cells can produce this protein in large quantities and express it over the entire membrane surface when losing their polarity. As these cells no longer rest on a basal lamina, CEA can be found in high concentrations in the blood of human patients with certain tumours.^15^ In healthy dogs, serum CEA concentrations reference values were initially set using radio‐immuno assays, ranging from 0.12 to 0.23 ng/ml,^16^, ^17^ and interestingly, bitches with mammary tumours presented CEA serum concentration above the reference values. At the 0.23 ng/ml cut‐off concentration, the sensibility and specificity of this assay was of 60% et 95% respectively for tumour diagnosis in dogs.^17^ Other studies evaluated the difference in CEA expression between female dogs with mammary tumours and healthy dogs using ELISA kits available for human CEA. Two studies confirmed a significant increase in serum CEA concentration in bitches with mammary tumours compared with healthy bitches^18^, ^19^ whereas a previous one showed no correlation of the CEA serum levels with the staging of the tumour.^20^ Interestingly, Senhorello et al., observed a significant increase in CEA levels in bitches with tumours greater than 3 cm in size compared with tumours less than 3 cm, and a significant increase in CEA was noticed for grade III tumours compared with grade I and II tumours. Based on the Receiver Operating Characteristic (ROC) curve performed using 1.08 ng/ml as the cut‐off value, they established the sensitivity and specificity of serum CEA, measured by ELISA, to detect the presence of mammary tumour at 82.14% and 95.24%, respectively. Sensitivity for this threshold value was improved to 100% for tumours larger than 3 cm and metastasised tumours but down to 70% for tumours smaller than 3 cm.^19^ Taken together, these studies confirmed that CEA is a relatively sensitive and specific diagnostic biomarker for canine mammary tumours, especially in more advanced stages, which, however, might not allow CEA to be a biomarker for early diagnostic of breast tumour. The use of human ELISA kits, commercially available to measure CEA in dog blood, made these studies possible but more recently, canine specific kits are also available, which is a great advantage to acknowledge CEA as an easily accessible canine cancer biomarker. These more recent studies are in agreement with what had been shown previously.^21^, ^22^ Using canine specific CEA ELISA kits, lower CEA levels were observed 15 days after surgery on 41 dogs but this difference was not significant.^21^ Interestingly, with human CEA ELISA kits, the reduced CEA serum levels were significantly different 15 and 45 days post‐masectomy as compared with before surgery in 11 female dogs.^19^ In terms of prognostic biomarkers, they also show that the higher the initial serum CEA levels in bitches, the shorter the survival time is,^21^ but longer‐term studies involving a larger number of bitches are required to assert this.

### 3.2 The carbohydrate antigen 15‐3

The Carbohydrate Antigen 15–3 (CA15‐3) is transmembrane glycoprotein belonging to the mucin family produced by the *MUC‐1* gene. The products of the *MUC‐1* gene are involved in carcinogenesis as they participate to immunosuppression,^23^ promote the proliferation and survival of tumour cells,^24^ and also contribute to their dissemination.^25^ CA15‐3 concentration is increased in blood when tissues are altered, however, this is not specific to a particular neoplastic disease and can be found in different human cancers^26^, ^27^, ^28^, ^29^, ^30^, ^31^ as well as in hepatitis^32^ and arthritis.^33^

In veterinary oncology, several studies have found a significant increase in serum CA15‐3 concentration in female dogs suffering from canine mammary tumours compared with healthy ones.^17^, ^18^, ^20^, ^21^, ^22^, ^34^, ^35^, ^36^ The sensitivity and specificity of CA15‐3 to detect mammary neoplastic damage at a threshold value of 7 IU/ml, was 100% and 95% respectively.^17^ However, a more recent study exhibited a sensitivity of CA15‐3 to 51.8% in the malignant group but with a specificity close to previously described (93.9%). More interestingly, combining CA15‐3 to CEA for instance, increased sensitivity to 64.2%, but combining these two biomarkers reduced specificity down to 81.7%.^22^ In addition, a significant positive correlation between increasing serum CA15‐3 concentrations and advancing stages of tumour has been observed.^20^ However, the preliminary study from 2007 by Marchesi et al., showed no significant difference in serum CA15‐3 concentrations between dogs with different types of neoplastic processes and healthy dogs.^34^ This lack of significance could be explained, as in humans, by the fact that not all tumour types are associated with an elevated CA15‐3 production. Finally, as for CEA, low serum CA15‐3 concentrations before surgery are associated with longer survival times, while higher concentrations are associated with shorter survival times.^21^ Thus, the value of CA15‐3 may provide prognostic information in bitches with mammary tumours. Now, studies showing the independence of this parameter from those used in practice for prognostic purposes are required to recommend serum CA15‐3 in prognostic procedures. Combining CA15‐3 with other biomarkers, as suggested above,^22^, ^37^ could also be a lead to follow for canine cancer prognosis.

### 3.3 The alpha‐fetoprotein

The alpha‐fetoprotein (AFP) is synthesised in large quantities mainly by the liver, during embryonic development. It is indeed one of the major proteins in foetal circulation, however, its expression is transcriptionally repressed at birth. AFP has been shown to have an immunosuppressive activity and also pro‐angiogenic properties that promote neovascularisation in foetal and tumour tissues.^38^, ^39^, ^40^ Considering that AFP is mainly produced by liver, veterinary studies have focused on this protein as a biomarker for canine liver cancer.

At birth, serum AFP concentration in dogs is high: 14080 ± 5944 μg/ml, then a strong decrease during growth is observed until reaching 0.014 to 0.069 μg/ml in adult dogs.^41^ In vitro, AFP can be produced by canine hepatocellular carcinoma cells, suggesting that this production could be linked to the neoplastic process in dogs.^42^ This observation had been confirmed by Marchesi et al. 2007, in which, no significant differences were noted in the serum AFP concentrations between healthy dogs and those with non‐neoplastic diseases but dogs with lymphomas and mastocytomas showed an increase in serum AFP concentrations. However, AFP concentrations in serum of dogs with sarcomas and carcinomas were not significantly different from healthy dogs.^34^ A high AFP serum concentration cut‐off value could indicate a high probability of neoplastic process.^43^, ^44^ but to date, no studies have proposed a cut‐off value in dogs defining a sensitivity and specificity value for this biomarker. Moreover, no studies have been conducted on hepatocellular carcinomas in dogs to establish a correlation between serum AFP concentration and histological characteristics, tumour size, or tumour stage. Despite a significant decrease in serum AFP concentrations observed following surgical removal of affected liver lobes,^43^, ^44^ the available evidence is not strong enough yet to consider AFP serum concentrations as a good follow‐up biomarker of individuals with hepatocellular carcinoma.

### 3.4 Lactade dehydrogenase

Rapid cancer cell proliferation and high metabolic demands lead to an increase in lactate dehydrogenase (LDH), which catalyses the reversible transformation of pyruvate into lactate and is well established in human medicine as a prognosis and follow‐up biomarker in many cancers.^45^ It has been shown in veterinary medicine that LDH is significantly increased in blood of dogs with malignancies compared with healthy dogs or dogs with non‐tumour disease and the highest LDH concentration is observed among dogs with lymphoma.^46^ This enzyme is easily measurable in freshly collected serum using commercially available kits and reference values in dog serum are set from 45 to 233 U/L.^20^ Interestingly, a significant increase of serum LDH in dogs with malignant mammary tumours has been measured compared with healthy dogs and a positive correlation was shown between LDH serum concentrations and tumour stage.^20^ Therefore, serum LDH concentration shows an interesting diagnostic value for canine tumours, despite an earlier study showing that dogs affected with malignant lymphoma kept LDH levels in the reference values.^47^ In cats, a study shows a poor accuracy for serum LDH to discriminate oral lymphoma and inflammatory bowel disease (IBD)^48^ and LDH levels were also high in serum of dogs infected with canine parvovirus.^49^ However, interestingly, an increased LDH activity (> 280 U/L) anticipated clinical stages of lymphomas in dogs and was also often seen at completion of chemotherapy and at 1 month after chemotherapy, in dogs with recurrence during the successive 45 days, with a sensitivity of 77.8% and 96.2% of specificity.^50^ Thereby, rather than a diagnostic biomarker, LDH could be an interesting prognostic tool to predict recurrence and a follow‐up biomarker in veterinary medicine, but further studies are needed to assess this hypothesis.

### 3.5 The anti‐mullerian hormone

The anti‐mullerian hormone (AMH) is a glycoprotein secreted by Sertoli cells in males and by the granulosa cells of small growing follicles in females. A decrease of its secretion has been observed after puberty in men, bulls and stallions.^51^ Thus, this hormone is mainly studied and used in relation to its potential as a biomarker in granulosa cell tumours (GCT) and Sertoli cell tumours (SCT) in humans.^52^ Given the importance of reproduction in veterinary breeding, blood AMH concentrations in the case of ovarian pathologies were first investigated in cows and mares. It was shown in both animals that an increase in the serum AMH concentration above the threshold limit diagnostics the presence of GCT, with the cut‐off set at 0.36 ng/ml for cows and 4 ng/ml for mares, with sensitivity respectively evaluated at 100% and 98%.^53^, ^54^ AMH serum concentrations were significantly higher in female dogs with GCT compared with healthy bitches as well as bitches with other ovarian neoplasia or non‐neoplastic ovarian disease such as follicular or luteal cysts. The cut‐off value was set at 0.99 ng/ml, which gives to AMH a sensitivity of 100% and specificity at 94.44% to detect GCT.^55^ These values suggest accurate diagnostic performance for AMH, but further studies would be required in bitches, including a larger number of animals, to confirm these results. Interestingly, in male dogs, several articles have also shown a threefold or more increase in serum AMH concentrations in SCT dogs compared with healthy dogs.^56^, ^57^ These results strengthened AMH as a potential biomarker for SCT in male dogs as well. However, the diagnostic interest of this marker is limited by the fact that any pathology concerning the ovaries or testicles is mostly treated by surgical excision in veterinary medicine. Therefore, a precise diagnostic test to detect a GCT or SCT before surgical removal is rarely necessary. Further studies are now needed to establish the potential clinical utility of this marker in the follow‐up of animals with GCT or SCT, including its ability to detect metastatic processes after surgical excision.

### 3.6 The thymidine kinase 1

The thymidine kinase 1 (TK1) is one isoform of the thymidine kinase (TK) which phosphorylates thymidine, an essential nucleotide for DNA replication. TK1 is located in the cytosol where its quantity and activity are low when the cell is quiescent and increase during cell division.^58^ Many studies in dogs have focused on TK1 as a biomarker in oncology. In most of these studies, it has been shown that the activity of TK1, measured by radioenzymatic assays or ELISA, is increased particularly in the blood of dogs suffering from lymphomas and myeloid leukaemia compared with healthy dogs.^47^, ^59^, ^60^, ^61^, ^62^, ^63^, ^64^, ^65^, ^66^ The diagnostic performances of TK1 activity, outlined by some of these authors, to differentiate dogs with haematopoietic neoplastic processes from healthy dogs is shown in Table 1. Thus, the activity of TK1, significantly increased in haematopoietic tumours, may be interesting in terms of diagnostic performances mainly for these specific tumours. In terms of prognostic interest, it has been shown that the activity of TK1 increased with tumour stage.^60^, ^65^ In addition, an increase in TK1 activity above 30 U/L is significantly associated with a decrease in overall survival time, in dogs with lymphomas^60^ and more recent studies showed that a combination of TK1 with the inflammation marker C Reactive Protein (CRP) gave higher diagnosis accuracy and prognostic values than either of both alone, in dogs with haematological malignancies.^65^, ^67^ Finally, several studies have investigated the interest of TK1 as a pharmacodynamic biomarker in dogs with haematopoietic tumours (lymphomas and leukaemias) during treatment. All these studies consistently showed that dogs undergoing chemotherapy had a significant decrease in TK1 activity, with partial or complete response, and up to physiological values.^60^, ^61^, ^64^, ^66^, ^68^, ^69^ According to these same studies, these values remained significantly higher in cases of relapse or no response to treatment. Thus, TK1 shows good values in terms of diagnostic, prognostic and follow‐up performance in canine haematopoietic tumours, and therefore seems to be a good candidate as a biomarker in this field, despite a certain lack of specificity of this enzyme for other neoplastic pathologies. Interestingly, the stability of TK1 enzyme activity is not affected by storage at −20°C for up to 3 month^60^ and has also been correlated with ELISA values in dogs with malignant lymphomas,^47^ therefore TK1 measurement could easily become a routine procedure in veterinary oncology.

**TABLE 1:** Diagnostic performances of TK1 in dogs with haematopoietic cancers (lymphomas and leukaemias) compared with healthy dogs^a^

Study	Dogs with neoplasia (*n*)	Healthy dogs (*n*)	Used technology	Sensitivity	Specificity
Nakamura et al., 1997^60^	20	13	Radioenzyme assay	100%	100%
von Euler et al., 2004^61^	52	21	Radioenzyme assay	92%	98.1%
Thamm et al., 2012^64^	14	24	ELISA	80%	n.d.
Sharif et al., 2012^65^	34	35	Radiochemical assay	94%	68%
Jagarlamudi et al., 2015^67^	43	42	ELISA	79%	97%

Abbreviation: n.d., non‐determined.

^a^ Calculated by authors from data in the articles.

## 4 NUCLEIC ACID BIOMARKERS

### 4.1 Circulating deoxyribonucleic acids

Increased concentrations of cell‐free circulating Deoxyribonucleic Acids (cell‐free DNA or cfDNA) have been observed in blood of human cancer patients as compared with healthy individuals. However, this increase has been shown later not to be specific of tumours since cfCDNA is also increased in other human conditions such as pregnancy or after transplantation and in some inflammatory conditions.^70^ Whereas cfDNA is the total pool of circulating cell‐free DNA, circulating‐tumour DNA (ctDNA) is tumour‐specific, released from circulating tumour cells (CTCs) and from cells within the tumour. Several studies in humans suggest that cells within the tumour are the main source of ctDNA compared with CTCs since ctDNA has been detected even in the absence of CTCs in the blood.^71^, ^72^ The ctDNA forms a very small part of the total circulating cfDNA, so it is particularly complex to detect. Somatic mutations (point mutations, insertions, rearrangements), variability in the number of copies of a gene, size of the fragment, aneuploidy and degree of methylation are used to identify ctDNA among cfDNA, using many different methods.^73^, ^74^ Furthermore, it was shown that, thanks to different properties of the ctDNA, such as its methylation profile, it was possible to determine the tissue of origin of the identified ctDNA.^75^, ^76^, ^77^ Thus, a combination of somatic and epigenetic changes could detect a tumour process and determine its location.

A limited number of studies have been carried out on cfDNA in veterinary oncology rather than on ctDNA. Most of the studies investigating the variation in the amount of cfDNA in dogs with neoplastic processes have shown a significant increase in the amount of cfDNA in patients compared with healthy dogs as shown in Table 2.^78^, ^79^, ^80^ Interestingly, Tagawa et al., show that the amount of plasma cfDNA is correlated with clinical stage, with a significant increase in plasma cfDNA in metastatic versus non‐metastatic dogs.^78^ In 2013, a first study carried out the complete sequencing of the tumour genome of four dogs with mammary tumours. Based on the genetic rearrangements observed within the tumours, the authors were able to detect in the four dogs, circulating DNA with these same rearrangements, that is, tumour circulating DNA.^81^ The specific *BRAF* gene mutation was also investigated in dogs with urothelial carcinomas since it had been shown that nearly 80% of these tumours in dogs have a mutation in this gene.^82^ The *BRAF* V595E mutation was found in almost 73% of the plasma of dogs with tumour and the concentration increased with disease progression. Thus, the authors suggest that the amount of ctDNA with *BRAF* mutations may represent a useful non‐invasive diagnostic biomarker in canine urothelial carcinoma.^83^ Interestingly the *BRAF* V595E mutation can also be monitored in urine samples using the new droplet digital Polymerase Chain Reaction (dPCR) molecular technique which allowed to identify the mutation in 83% of dogs with urothelial carcinoma (19 positive out of 23 patients) and 100% of dogs with protastic carcinoma (3 dogs only) when it was completely absent in healthy controls.^84^

**TABLE 2:** Studies comparing cfDNA quantities in neoplastic and healthy dogs

Study	Tumour group		Healthy group		*p‐*value^a^
	Type and number of tumour bearing dogs	[cfDNA]^b^ in blood	Number of healthy dogs	[cfDNA]^b^ in blood	
Letendre and Goggs, 2017^81^	Sarcomas *n* = 15	8.1 μg/ml^c^ (5.1–15.8)	*n* = 15	4.7 μg/ml^c^ (0.6–8.5)	*p* = .003
Beffagna et al., 2017^82^	Mammary tumours *n* = 44	Short fragments: 128.5 ng/ml (56.8–200.2) Long fragments: 87.5 ng/ml (33–142)	*n* = 15	Short fragments: 28.8 ng/ml (6.5–51.1) Long fragments: 18.2 ng/ml (3.9–32.6)	Short fragments: *p* = .018 Long fragments: *p* = .009
Tagawa et al., 2019^80^	Various tumours^d^ *n* = 50	6290 ng/ml (51–115 000)	*n* = 11	481 ng/ml (205–802)	*p* = .011

^a^ In all studies, *p* < .05 is considered significant.

^b^ [cfDNA]: concentration of cell‐free DNA.

^c^ Median values and when non‐specified mean values.

^d^ Lymphoma & leukaemia (*n* = 11), hemangiocarcinoma (*n* = 8), urothelial carcinoma (*n* = 5), mammary carcinoma (*n* = 4), orar squamous cell carcinoma (*n* = 3) apocrine gland anal sac carcinoma (*n* = 3) hepatic tumour (*n* = 3), soft tissue carcinoma (*n* = 2), malignant melanoma (*n* = 2), gastrointestinal stroma tumour (*n* = 2), mast cell tumour (*n* = 1), osteosarcoma (*n* = 1), ceruminous adenocarcinoma (*n* = 1), salivary adenocarcinoma (*n* = 1), apocrine adenocarcinoma (*n* = 1), pheocromocytoma (*n* = 1), seminoma (*n* = 1).

Another specific element of ctDNA is the hypomethylation of the Long Interspersed Nuclear Element‐1 (LINE‐1) sequences which are associated with oncogenesis and metastases formations.^85^ In a study involving healthy dogs, dogs with malignant or benign cancers, the index of the relative level of methylation of the LINE‐1 sequences of the cfDNA was significantly reduced in dogs with benign and malignant mammary tumours (0.29 ± 0.061 and 0.39 ± 0.066, respectively) compared with healthy dogs (0.92 ± 0.067). However, this decrease was less significant for other tumour types studied in this article.^86^

cfDNA and ctDNA appear to be of interest as well as prognostic and follow‐up biomarkers in dogs with neoplastic processes. Indeed, dogs with high cfDNA concentrations had a significantly shorter duration of remission/lower overall survival rate than dogs with low cfDNA concentration with a threshold set at 25 ng/ml when the reference value for cfDNA in normal canine plasma is 1–15 ng/ml.^87^ Looking more specifically at ctDNA, two other studies came to the same conclusions.^83^, ^88^

Despite the low number of studies in dogs, cfDNA and ctDNA as diagnostic, prognostic and monitoring biomarkers in patients suffering from neoplastic processes should be considered. The advantages of measuring cfDNA and/or ctDNA are numerous: it can be detected in blood as well as in non‐invasive samples such as urine, appears reliable in detecting a tumour process in a sensitive and specific manner, and allows the entire tumour process of an individual to be properly characterised. However, studies involving a larger number of dogs are needed to establish reliable results and to be integrated into clinical practice in veterinary oncology.

### 4.2 Circulating ribonucleic acids

As for DNA, RNA can also be found in the bloodstream. The main circulating RNAs are microRNAs (miRNAs). Highly conserved in evolution, they act as post‐transcriptional regulators, based on the principle of partial or complete correspondence with the complementary mRNA. To date, several thousand miRNAs have been identified in humans.^89^ Several studies have been conducted on miRNAs as a biomarker in dogs, but only a few have focused on their expression in the blood of dogs with neoplastic processes. *In vitro*, the miRNA profiles contained in exosomes released from normal canine mammary epithelial cells obtained from dogs without mammary tumours were compared with exosomes released by tumour breast cells, derived from canine mammary carcinoma. A total of 145 miRNAs were differentially expressed by tumour cells compared with healthy cells.^90^ However, two other studies in dogs concluded that there was no correlation between the miRNA profiles recorded within the tumour and in the blood of the same patient.^91^, ^92^ Thus, the increases or decreases in blood concentrations recorded for some miRNAs, may not be due solely to differential expression of the miRNAs by the tumour, but may be the result of a general response of the body to the presence of the tumour. Nevertheless, some studies focused on specific miRNA, such as miRNA‐126 and ‐214, which are known to play a role in angiogenesis and tumorigenesis.^93^ Two studies showed a significant increase in blood expression of both biomarkers in dogs with hemangiosarcoma, compared with dogs with benign processes and healthy dogs. It should also be noted that the values of these two markers did not show a strong correlation with other clinical parameters (haematocrit, platelet count, prothrombin time and activated partial thromboplastin time, blood urea nitrogen, creatinine, alanine aminotransferase, aspartate aminotransferase, alkaline phosphatase), thus attesting to the independent nature of these biomarkers.^93^, ^94^ Interestingly, as in human medicine where a panel of several miRNA showed very high values of diagnosis accuracy to detect people with early stage of non‐small‐cell‐lung‐tumours compared with either miRNA alone,^95^ a combination of miRNA‐214 and miRNA‐126 has showed better diagnosis accuracy to detect different types of epithelial and non‐epithelial tumours than both independently.^93^, ^94^ Several additional studies have looked at other mi‐RNAs, particularly with regard to specific tumour conditions. Serum miRNA‐122 concentrations have been shown to be increased in dogs with neoplastic liver damage compared with healthy dogs.^96^ RNA deep‐sequencing (RNASeq) and dPCR techniques identified differential expression of 65 miRNAs in the blood of healthy female dogs and of dogs with mammary carcinomas, with two of them significantly over‐expressed in the plasma of dogs with tumours: miRNA‐19b and miRNA‐125a. In addition, the plasma concentration of miRNA‐18a was significantly increased in dogs with Stage III versus Stage I–II tumours. A significant increase in plasma miRNA‐18a concentration was also observed when lymphatic invasion was noticed on histological analysis, and could therefore be a predictive biomarker of metastasis.^97^

Regarding the prognostic performance of blood miRNAs in dogs, an increase in serum miRNA‐125a concentration showed a correlation, although not significant, with a decrease in survival time in bitches with mammary carcinoma.^97^ Moreover, combined miRNA‐126 and ‐214 concentrations did not show a significant impact on survival times of dogs affected by various type of neoplasia.^93^ A study focusing on the prognostic impact of miRNA‐126 and ‐214 concentrations in dogs with osteosarcoma treated by amputation and adjuvant chemotherapy, revealed that an increase in serum miRNA‐214 concentration was significantly associated with poorer duration of remission and shorter overall survival time. Higher miRNA‐126 concentrations were on the contrary, significantly associated with longer durations of remission and overall survival. It should be noted that the values of these markers were independent of different histopathological characteristics (mitotic index, histological subtype), but an association was noted with tumour grade.^98^ For dogs with multicentric lymphomas, elevated plasma miRNA‐21, ‐31, ‐45, ‐150, ‐155 and ‐222 concentrations were significantly correlated with lower progression‐free survival rates, and elevated plasma miRNA‐181c and ‐222 concentrations significantly associated with a lower median overall survival time but in the B‐cell lymphoma group only.^92^ Thus, several circulating miRNAs appear to have shown potential as prognostic biomarkers in dogs with different tumour types, although few studies have been performed on this subject.

Interestingly, serum miRNA‐126 and ‐214 concentrations were decreased in dogs after surgical removal of a hemangiosarcoma, attesting the dynamic nature of these two miRNAs.^94^ Some other miRNAs also showed significant variation in their serum concentrations in dogs with lymphomas during relapses (increased miRNA‐125b but miRNA‐30b, ‐34a and ‐182 decreased).^92^ These observations could trigger an interest to use these miRNAs for monitoring tumour processes in dogs and early detection of relapse. However, it is important to underline that a lack of specificity of miRNAs in neoplastic condition could be a main limitation for these as diagnostic biomarkers. Indeed, for example, there were no significant differences in plasma concentrations of miRNA‐122 between dogs with hepatic neoplasia, hepatic fibrosis or inflammatory liver disease.^96^ Moreover, some miRNA detected in the blood such as miR‐144 or miR‐32, are not able to discriminate dogs with metastasis from dogs without,^91^ adding one more limitation to their value as prognostic biomarkers.

In conclusion, some specific circulating miRNAs have been shown in humans and dogs to be of interest for diagnosis, prognosis or prediction of responses to treatment and for monitoring individuals with neoplastic processes. For all these reasons, miRNAs are presented as the future major biomarkers in the field of oncology by many authors and more studies on these in veterinary oncology can be expected in the future.

## 5 CIRCULATING CELLS AS BIOMARKERS

### 5.1 Circulating tumour cells

Circulating Tumour Cells (CTCs) are responsible for the formation of metastases. CTCs originate from a solid tumour from which they have lost their ability to adhere, allowing them to migrate into the blood. As these cells are very rare, about 1 CTC among 10^6^ to 10^7^ leukocytes, the tools used for their detection must be highly sensitive and techniques for enrichment and isolation of these cells, are necessary.^99^, ^100^ They are based on the recognition of CTCs' cell surface markers, on their physical or biological characteristics (density, size, invasiveness), or on molecular techniques such as RT‐PCR for the detection of CTCs‐specific RNA. However, only one technique for identifying, isolating and enumerating CTCs was approved in 2004 by the Food and Drug Agency (FDA) which includes an enrichment step followed by a more specific identification, both using specific antibodies and high resolution imaging systems.^100^ The cells considered as CTCs express the Epithelial cell adhesion molecule (EpCAM), Cytokeratins (CK), and are positive for 4′,6‐diamidino‐2‐phenylindole (DAPI) but do not express the Cluster of Differentiation 45 (CD45). Their phenotype is therefore EpCAM+/CK+/DAPI+/CD45–.^101^ Due to the fact that this is the only FDA‐approved technology, the majority of studies in human oncology have been conducted using this system, but in dogs, studies have been published using RT‐PCR as it is possible to detect the presence of mRNAs specifically expressed in CTCs in the blood.^102^ Out of 16 human breast cancer CTCs markers mRNAs, six were overexpressed in the blood of bitches with mammary carcinoma but none were in the blood of healthy dogs.^103^, ^104^ Moreover, products of the *CLDN7*, *CRYAB*, *ATP8B1* and *EGFR* genes can differentiate with varying degrees of accuracy between bitches with metastatic breast carcinoma and bitches with no evidence of metastasis. In particular, mRNAs from the *CRYAB* and *ATP8B1* genes could be biomarkers for CTCs in female dogs with mammary carcinoma, since they both exhibit respectively the best sensitivity and specificity values (35% and 100% for *CRYAB* and 32.5% and 90% for *AT8B1*) among all the other genes tested.^105^

EpCAM+/CK+/DAPI+/CD45‐ CTCs have also been identified in the blood of bitches with metastatic mammary carcinoma using the FDA approved method for human samples. This new technique showed a sensitivity of 44.4% for the detection of metastasized mammary carcinoma, and revealed that the presence of CTCs in the blood has a negative prognostic impact.^106^ Recently, flow cytometry was also used to detect CTCs in the blood of three dogs with osteosarcoma with labelling of Collagen I and Osteocalcin. Interestingly, CTC frequencies in blood were greatly reduced following amputation in all three dogs, were variable during chemotherapy and an increase has been seen in all three dogs within the 4 weeks prior to apparent metastasis or death.^107^ This first study showed a dynamic evolution of CTCs during chemotherapy treatment in dogs, suggesting a potential follow‐up biomarker role for CTCs in dogs with osteosarcoma during treatment.

In conclusion, CTCs have shown their potential for diagnosis of tumour processes despite limited and variable sensitivity. They have also proven to be interesting in terms of prognosis, monitoring and predictive tools. However, given that flow cytometry and CTCs research is a relatively recent field in veterinary oncology, it is likely that the increasing number of studies will lead in the near future to a wider use of these cellular biomarkers.

### 5.2 Myeloid derived suppressor cells

Myeloid derived suppressor cells (MDSCs) form a group of heterogeneous cells mainly present in the bone marrow where they represent the immature stages of granulocytes and macrophages. They have a strong immunomodulatory activity by mainly inhibiting the proliferation of T lymphocytes, reducing their cytotoxic power and inducing their apoptosis. They also reduce the proliferation and activity of B lymphocytes, as well as natural killer (NK) cells and dendritic cells. Acute or chronic inflammatory phenomena favour the accumulation of MDSCs within the sites of inflammation, prevent their differentiation and induce their activation.^108^ It has been shown that many factors allow the recruitment and expansion of MDSCs into the tumour environment from the bone marrow and secondary lymphoid organs. Due to their immunosuppressive role and their angiogenic capacities, human MDSCs are key players in tumour escape and metastasis formation.^109^ A significant increase in the proportion of MDSCs among peripheral blood mononuclear cells (%MDSCs) has been shown in a wide range of tumour types compared with the %MDSCs in healthy human subjects.^110^ Thus, due to the large number of tumour types with a significant increase in %MDSCs, this endpoint cannot represent a diagnostic biomarker of a particular tumour but may indicate the possibility of a tumour involvement. MDSCs were first identified in dog blood by flow cytometry as immature myeloid cells expressing CD11b, but lacking the expression of CD14 and the major histocompatibilty complex class II (MHCII).^111^ The immunosuppressive character of these cells was confirmed by co‐culture experiments in the presence of autologous or heterologous T lymphocytes, whose activity was reduced in the presence of MDSCs. Blood of dogs with advanced neoplastic processes showed a significant increase in %MDSCs (36.04%) compared with healthy dogs (10.24%) and early‐stage tumours (9.40%).^111^ These results were confirmed by other studies on canine MDSCs in the context of sarcoma, carcinomas and oral melanomas^112^ and mammary tumours.^113^, ^114^ It was proposed later, that canine MDSCs could be distinguished, as in humans and mice, in two different phenotypes: monocyte‐derived MDSCs (M‐MDSCs) and polymorphonuclear‐MDSCs (PMN‐MDSCs) by flow cytometry, microscopy, and co‐culture experiments. Both cell populations are CD5−/CD21−/MHCII− and CD11b+ and they differ from each other by the expression of CD14 and CADO48A, a canine specific neutrophil marker. M‐MDSCs are CD14+/CADO48A−, whereas PMN‐MDSCs are CD14−/CADO48A+. The percentage of PMN‐MDSCs among blood mononuclear cells (%PMN‐MDSC) was increased in dogs with tumour processes compared with healthy dogs, and this difference was significant in some tumour types.^115^ In dogs with melanomas, a significant increase in %M‐MDSC and %PMN‐MDSC compared with healthy dogs was also observed.^116^ In humans, MDSCs have shown a moderate diagnostic capacity, with modest sensitivities and specificities for the detection of tumour processes.^117^, ^118^, ^119^ However, the prognostic capacities of blood %MDSCs have been widely demonstrated, as well as their ability to predict the response to treatments based on their initial levels. However, although their increase is more pronounced for advanced neoplastic processes, their prognostic impact has not been clearly established in dogs with tumours. Finally, their usefulness in terms of follow‐up during treatment could not yet be clearly established in both humans and dogs. Therefore, additional studies are necessary to confirm the results obtained, to study the prognostic impact of MDSCs and their usefulness in the follow‐up of animals under treatment.

## 6 CONCLUSION

In this review, we focused on blood biomarkers studied in veterinary oncology, and that shows a certain degree of specificity and sensitivity for neoplastic processes. Most of the biomarkers highlighted to date are protein‐based, including CEA, CA15‐3, AFP, LDH, AMH and TK1. They all show varying performances in terms of sensitivity and specificity to tumour, and each has shown some evidence of their potential as diagnostic, prognostic, predictive and monitoring biomarkers. However, none of them has perfect specificity for distinguishing individuals with tumour processes from healthy individuals or those with non‐neoplastic pathologies. The next generation of biomarkers from liquid biopsy, seems to be particularly encouraging such as circulating miRNAs, ctDNA, cfDNA, CTCs and MDSCs. Some of these biomarkers have shown high performances in terms of sensitivity and specificity for identifying individuals presenting a tumour condition. It also appears that they have the capacity to describe tumour characteristics and monitor their evolution. More specifically, they allow to predict precisely sensitivities of the tumours studied to the various treatment protocols, both in humans and dogs. According to the One Health concept, most of the biomarkers studied in humans are also accurate to follow in dogs, and conversely dogs could also be a good model to identify or better characterise them, leading to a true personalised medicine, for both humans and dogs.

## AUTHOR CONTRIBUTIONS

**Philippe Colombe**: Acquisition of data, analysis, and interpretation of data, drafting and revising the manuscript. **Jérémy Béguin**: revising the manuscript. **Ghita Benchekroun**: revising the manuscript. **Delphine Le Roux**: conception and design, analysis and interpretation of data, drafting and revising the manuscript.

## FUNDING INFORMATION

The authors received no funding for this work.

## CONFLICT OF INTEREST

The authors have no conflict of interest to declare.
